# Stereocontrolled semi-syntheses of deguelin and tephrosin†
†Electronic supplementary information (ESI) available: ^1^H and ^13^C NMR spectra. See DOI: 10.1039/c6ob02659a



**DOI:** 10.1039/c6ob02659a

**Published:** 2017-01-24

**Authors:** David A. Russell, Julien J. Freudenreich, Joe J. Ciardiello, Hannah F. Sore, David R. Spring

**Affiliations:** a Department of Chemistry , University of Cambridge , Lensfield Road , Cambridge , CB2 1EW , UK . Email: spring@ch.cam.ac.uk

## Abstract

We describe stereocontrolled semi-syntheses of deguelin and tephrosin, anti-cancer rotenoids isolated from *Tephrosia vogelii*. Firstly, we present a new two-step transformation of rotenone into rot-2′-enonic acid *via* a zinc-mediated ring opening of rotenone hydrobromide. Secondly, following conversion of rot-2′-enonic acid into deguelin, a chromium-mediated hydroxylation provides tephrosin as a single diastereoisomer. An Étard-like reaction mechanism is proposed to account for the stereochemical outcome. Our syntheses of deguelin and tephrosin are operationally simple, scalable and high yielding, offering considerable advantages over previous methods.

## Abstract 2

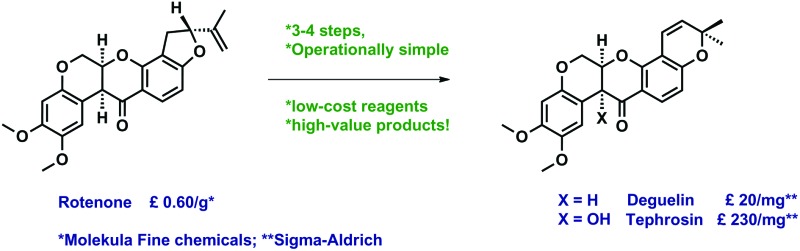
We describe stereocontrolled semi-syntheses of deguelin and tephrosin, anti-cancer rotenoids isolated from *Tephrosia vogelii*.

## Introduction

**Fig. 1:**
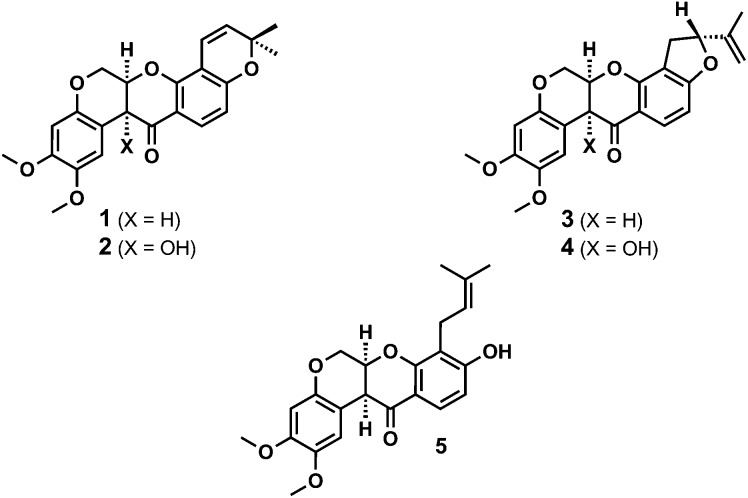
Structures of the rotenoids deguelin **1**, tephrosin **2**, rotenone **3** and rotenolone **4** and rot-2′-enonic acid **5**.

Natural rotenoids display a wide range of biological activities, from strong pesticidal and insecticidal activities to therapeutically intriguing anticancer properties.^1,2^ Deguelin **1** and tephrosin **2** (Fig. 1) were isolated from *Teprosia vegelii* by Hanriot in 1907 and immediately identified as the principal bioactive components of the plant.^3^ Clark subsequently deduced the skeletal structures of both substances between 1930 and 1932^4^ and their absolute stereochemistries were resolved by analogy with rotenone in 1961 through Crombie's inspired degradative work^5^ and Djerassi's optical rotatory dispersion studies.^6^

Over the past two decades deguelin **1**, in particular, has been shown to inhibit the viability, proliferation and migration of multiple cancer cell lines,^2^ including difficult to treat prostate cancer^2*i*^ and pancreatic cancers xenograft models.^2*j*^ Further, rationally designed analogues of deguelin **1** have been shown to disrupt the function of HSP-90, leading to inhibition of HIF-1α and induction of mitochondrial apoptosis.^7^ As such, the synthesis and biological evaluation of rotenoids remains an area of considerable interest and opportunity.

As part of an extensive series of studies on the rotenoid group underway in our laboratory we required gram-scale quantities of deguelin **1** and tephrosin **2**. While several impressive total syntheses of both natural products have been reported,^8^ we reasoned that shorter stereocontrolled semi-syntheses from rotenone **3**, available commercially in kilogram quantities, would be better suited to large-scale preparations.

**Scheme 1:**
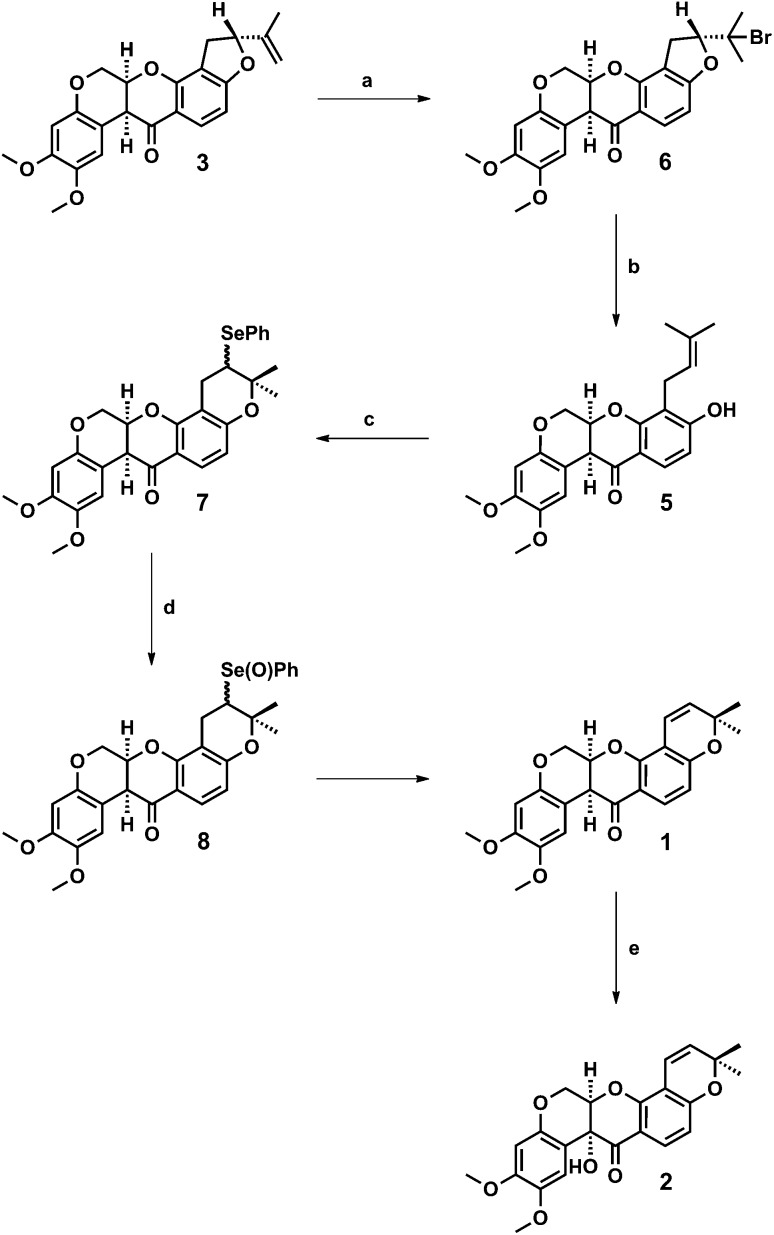
Reagents and conditions: (a) HBr, AcOH, rt, 0.5 h, 87%; (b) Zn, NH_4_Cl, THF, H_2_O, rt, 48 h, 79%; (c) PhSeCl, CH_2_Cl_2_, –40 °C, 2 h then rt, 1 h; (d) aq H_2_O_2_, THF, 0 °C, 1 h, then rt, 18 h, 81% from **5**; (e) K_2_Cr_2_O_7_, AcOH, H_2_O, 60 °C, 0.5 h then rt, 18 h, 76%.

A semi-synthesis of deguelin **1** from rotenone **3** (Fig. 1) was reported by Anzeveno in 1979,^9^ building upon Unai, Yamamoto and Crombie's earlier works on the selective E-ring cleavage of rotenone.^10,11^ The key intermediate in the synthesis was rot-2′-enonic acid **5** (Scheme 1), however its preparation involved the reductive dehalogenation of an allylic bromide with sodium cyanoborohydride in neat hexamethylphosphoramide.^9^

Consequently, we sought a new route to rot-2′-enonic acid **5** using less hazardous chemistry that could be more easily scaled-up to provide gram quantities of deguelin **1** following Anzeveno's cyclisation of rot-2′-enonic acid **5**.^9^ On the basis of Crombie's studies on the diastereoselective chromium-mediated hydroxylation of rotenone **3** to rotenolone **4**,^5*b*^ we reasoned that an analogous hydroxylation of deguelin **1** with potassium dichromate in aqueous acetic acid would provide tephrosin **2**.

## Results and discussion

First, we addressed the synthesis of rot-2′-enonic acid **5**, the synthetic and biosynthetic precursor to deguelin **1**, starting from rotenone **3**. A new two-step synthesis was devised in which a zinc-mediated ring opening of rotenone hydrobromide **6** afforded rot-2′-enonic acid **5** under mild conditions (Scheme 1).

The reaction of rotenone **3** with hydrogen bromide in acetic acid afforded rotenone hydrobromide **6** in 82–89% yield following its precipitation from the reaction mixture and crystallisation from chloroform–methanol. Best results were obtained with fresh reagent. Further, the reaction of rotenone hydrobromide **6** with excess activated zinc dust and ammonium chloride in aqueous THF proceeded smoothly to provide rot-2′-enonic acid **5** in 74–79% yield after extraction and crystallisation from methanol. Complete conversion of starting material was observed after 2 days. The use of commercial (un-activated) zinc dust afforded comparable conversion and yield after 4 days. In addition, the coagulated zinc that formed over the course of the reaction may be recovered (prior to extractive work-up) and reused. We were able to rapidly prepare more than 2.5 g of rot-2′-enonic acid **5** from 4 g (approximately 10 mmol) of rotenone **3** without the need for chromatography.

Having established a scalable route to rot-2′-enonic acid **5**, and with multiple grams of material in hand, we sought to complete the syntheses of deguelin **1** and tephrosin **2**. Treatment of rot-2′-enonic acid **5** with phenylselenyl chloride in dichloromethane at –35 °C, according to Anzeveno's established procedure,^9^ afforded an approximately 1 : 1 mixture of 5′-epimeric selenides **7** in quantitative yield. The crude selenides **7** were immediately oxidised with hydrogen peroxide in aqueous THF at 0 °C to their corresponding selenoxides **8**, which underwent spontaneous elimination upon warming to room temperature to afford deguelin **1** in 81% yield from rot-2′-enonic acid **5**.

Lastly, we were pleased to observe that the reaction of deguelin **1** with potassium dichromate in aqueous acetic acid proceeded smoothly to afford tephrosin **2** in 76% yield.

A mechanism for the dichromate hydroxylation of deguelin **1** is proposed in which we view the transformation as an Étard-like benzylic oxidation.^12^ Oxidation of an enol intermediate is discounted on the basis of the Crombie and Unai's extensive studies on the aerial oxidation of enolates derived from natural (optically active) and racemic rotenoids, which afford diastereoisomeric mixtures of *cis* and *trans* alpha-hydroxylated products.^5b,13^

**Scheme 2:**
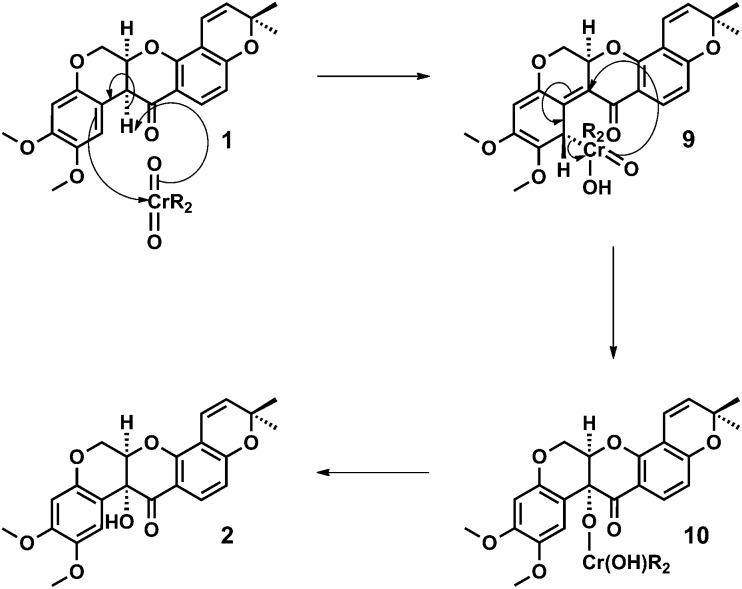
Proposed mechanism for the diastereoselective (stereocontrolled) Étard-like hydroxylation of deguelin **1** to tephrosin **2**, involving a facially selective ene reaction followed by a [2,3]-sigmatropic rearrangement and chromate ester hydrolysis.

In accordance with the Étard-like mechanism,^12*b*^ a necessarily diastereoselective (facially selective) ene reaction between deguelin **1** and the oxidant affords a stereodefined organochromium species **9** that subsequently undergoes a [2,3]-sigmatropic rearrangement to form a tertiary chromate ester **10**. Hydrolysis of the chromate ester upon work-up then affords tephrosin **2** as a single diastereoisomer. We would therefore attribute the diastereoselective outcome of the reaction to its doubly pericyclic nature, the necessary geometric requirements of these processes together with the characteristic ‘butterfly-wing’ architecture of the starting material (Scheme 2).

## Conclusions

We have developed stereocontrolled semi-syntheses of both deguelin **1** and tephrosin **2** starting from rotenone **3** and proceeding *via* rot-2′-enonic acid **5**. Firstly, a new transformation of rotenone **3** into rot-2′-enonic acid **5** is described that involves a zinc-mediated ring opening of rotenone hydrobromide **6**. This alternative preparation of rot-2′-enonic acid **5** avoids the use of the highly toxic reagents previously required in Anzeveno's synthesis^9^ and affords a higher yield across two steps (approximately 70% *vs.* 35%). The conversion of rot-2′-enonic acid **5** into deguelin **1** was achieved following Anzeveno's method.^9^ Finally, the transformation of deguelin **1** into tephrosin **2** was accomplished using a highly diastereoselective chromium-mediated hydroxylation, for which an Étard-like reaction mechanism is tentatively proposed.^12*b*^ Our syntheses provide deguelin **1** and tephrosin **2** in 56% and 42% yield respectively, involve only two chromatographic purifications and allow gram quantities of valuable enantiopure materials to be prepared simply and efficiently, facilitating biological studies thereof.

Lastly, we note that although commercially available at the time of writing, deguelin **1** and tephrosin **2** cost approximately 2 × 10^4^ and 2 × 10^5^ times more than rotenone **3**. We present operationally simple semi-syntheses of deguelin **1** and tephosin **2** starting from relatively inexpensive rotenone **3** using similarly low-cost reagents.
