# Supplementary material for: Stereocontrolled semi-syntheses of deguelin and tephrosin
Source: Org Biomol Chem. 2017 Jan 24;15(7):1593–6. doi: 10.1039/c6ob02659a (PMC5471929; doi:10.1039/c6ob02659a)
Supplement: Supplementary file 1 [file OB-015-C6OB02659A-s001.pdf]

Electronic Supplementary Material (ESI) for Organic & Biomolecular Chemistry.
This journal is © The Royal Society of Chemistry 2017

Electronic Supplementary Information:

Stereocontrolled Semi-Syntheses of Deguelin and Tephrosin

David A. Russell,[a] Julien J. Freudenreich,[a] Joe J. Ciardiello,[a] Hannah F. Sore[a] and David R. Spring*[a]

[a]Department of Chemistry, University of Cambridge, Lensfield Road, Cambridge, CB2 1EW, UK

*Email: spring@cam.ac.uk

Contents:

Experimental procedures

^{1}H and ^{13}C NMR Spectra for all compounds

Alternative version of Scheme 2

Experimental Procedures

General Methods

(6a*S*,12a*S*,5'*R*)-Rotenone **3** (95% purity) was purchased from Molekula Fine Chemicals as an off-white amorphous solid, which was crystallised from ethanol three times to give colourless plates (m.p. 162-163 ^{o}C, (lit. 163 ^{o}C)).[5a] All other solvents and reagents were used as obtained from commercial sources. Thin layer chromatography retention factors (R_f) are quoted to the nearest 0.05. Flash column chromatography was carried out according to the method of Still.[14] Yields refer to chromatographically and spectroscopically pure compounds, for which full analytical data are given. ^{1}H and ^{13}C NMR spectra were recorded on a Bruker Avance 500 Cryo Ultrashield (500 MHz) spectrometer at 298 K. Optical rotations were recorded on an Anton-Paar MCP 100 polarimeter. $[\alpha]_D$ values are reported in 10^{-1} deg cm^{2} g^{-1} at 598 nm, concentration (*c*) is given in g(100 mL)$^{-1}$. Melting points for crystalline compounds were obtained using a Büchi Melting Point B-545 melting point apparatus. Infrared spectra were recorded on a Perkin-Elmer Spectrum One spectrometer. Low-resolution mass spectra (LRMS) were recorded using an LCMS system (Agilent series LC with an ESCi Multi-Mode Ionisation Waters ZQ spectrometer using MassLynx 4.1 software). High-resolution mass spectra (HRMS) were recorded using a Micromass Q-TOF.

The nomenclature and numbering systems used for the assignments in this manuscript are well established in this natural product family.[1c] See also ESI Figure 1:

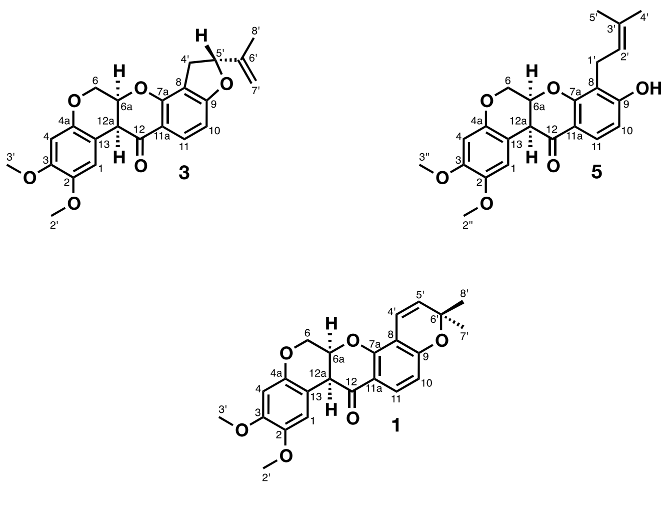

ESI Figure 1: Representative numbering schemes for rotenone **3**, rot-2'-enonic acid **5** and deguelin **1**.

(6a*S*,12a*S*,5'*R*)-Rotenone hydrobromide 6

Hydrogen bromide (32 mL, 33 % solution in acetic acid) was added dropwise over a period of 20 min to a suspension of (6a*S*,12a*S*,5'*R*)-rotenone **3** (4.00 g, 10.2 mmol) in acetic acid. The deep red reaction mixture was stirred at room temperature for a further 0.5 h (the product began to precipitate after approx. 10 min). Water (200 mL) was then added and the mixture was stirred vigorously for 0.5 h while an off-white precipitate formed, which was collected by filtration and dried *in vacuo* overnight. The precipitated solid was crystallised from chloroform-methanol to afford (6a*S*,12a*S*,5'*R*)-rotenone hydrobromide **6** as white needles (4.20 g, 87%). m.p. 127-128 ^{o}C; R_f 0.30 (hexane-ethyl acetate; 2:1); $[\alpha]_D^{20}$ -134 (*c* 0.1 in $CHCl_3$); ν_{max} (neat)/cm^{-1} 1676m (C=O)^{ketone}, 1608m, 1593m, 1502m, 1456m, 1344m, 1307m, 1263m, 1232m, 1213m, 1197m, 1092m, 1010m, 821m, 751m; δ_H (500 MHz, $CDCl_3$) 1.77 (3H, s, C(7')$\underline{H}_3$), 1.81 (3H, s, C(8')$\underline{H}_3$), 3.20 (1H, dd, *J* 8.0, 16.5 Hz, C(4')$\underline{H}$H'), 3.30 (1H, dd, *J* 9.5, 16.5 Hz, C(4')H$\underline{H}$'), 3.76 (3H, s, C(2')$\underline{H}_3$), 3.81 (3H, s, C(3')$\underline{H}_3$), 3.85 (1H, d, *J* 4.5 Hz, C(12a)$\underline{H}$), 4.19 (1H, d, *J* 12.0 Hz, C(6)$\underline{H}$H'), 4.63 (1H, dd, *J* 3.0, 12.0 Hz, C(6)H$\underline{H}$'), 4.74 (1H, dd, *J* 8.0, 9.5 Hz, C(5')$\underline{H}$), 4.95 (1H, ddd, *J* 1.0, 3.0, 4.5 Hz, C(6a)$\underline{H}$), 6.45 (1H, s, C(4)$\underline{H}$), 6.49 (1H, d, *J* 8.5 Hz, C(10)$\underline{H}$), 6.76 (1H, s, C(1)$\underline{H}$), 7.84 (1H, d, *J* 8.5 Hz, C(11)$\underline{H}$); δ_C (125 MHz, $CDCl_3$) 28.5 ($\underline{C}$(7')H_3), 30.1 ($\underline{C}$(4')H_2), 30.5 ($\underline{C}$(8')H_3), 44.6 ($\underline{C}$(12a)H), 55.9 ($\underline{C}$(3')H_3), 56.3 ($\underline{C}$(2')H_3), 65.8 ($\underline{C}$(6')), 66.2 ($\underline{C}$(6)H_2), 72.2 ($\underline{C}$(6a)H), 91.2 ($\underline{C}$(5')H), 100.9 ($\underline{C}$(4)H), 104.7 ($\underline{C}$(10)H), 104.7 ($\underline{C}$(13)), 110.3 ($\underline{C}$(1)H), 112.8 ($\underline{C}$(8)), 113.5 ($\underline{C}$(11a)), 130.0 ($\underline{C}$(11)H), 143.7 ($\underline{C}$(2)), 147.4 ($\underline{C}$(3)), 149.5 ($\underline{C}$(4a)), 157.7 ($\underline{C}$(7a)), 167.0 ($\underline{C}$(9)), 188.9 ($\underline{C}$(12)); LRMS *m/z* found 475.0 and 477.0, $C_{23}H_{24}O_6{}^{79}Br$ $[M + H]^+$ requires 475.1 and $C_{23}H_{24}O_6{}^{81}Br$ $[M + H]^+$ requires 477.1; HRMS *m/z* found 475.0768 and 477.0737, $C_{23}H_{24}O_6{}^{79}Br$ $[M+H]^+$ requires 475.0756 and $C_{23}H_{24}O_6{}^{81}Br$ $[M+H]^+$ requires 477.0736.

(6a*S*,12a*S*)-Rot-2'-enonic acid 5

Activated zinc dust (5.47 g, 84.2 mmol) was added to a suspension of (6a*S*,12a*S*,5'*R*)-rotenone hydrobromide **6** (4.00 g, 8.42 mmol) and ammonium chloride (1.80 g, 33.7 mmol) in THF (96.0 mL) and water (16.0 mL). The reaction mixture was stirred at room temperature for 48 h. The reaction mixture was then decanted from the coagulated zinc; diethyl ether (200 mL) was added, followed by water (200 mL) and aqueous hydrochloric acid (3.0 M, 10 mL). The two phases were mixed vigorously for 0.5 h. The organic phase was separated, washed with brine (200 mL), dried over anhydrous $MgSO_4$, filtered and concentrated *in vacuo*. The crude pale yellow solid obtained was crystallised from methanol to afford (6a*S*,12a*S*)-rot-2'-enonic acid **5** as white needles (2.63 g, 79%). m.p. 206-208 ^{o}C from methanol (lit. m.p. 207-208 ^{o}C from methanol); R_f 0.15 (hexane-ethyl acetate; 2:1); $[\alpha]_D^{20}$ +29 (*c* 0.1 in $CHCl_3$) (lit. $[\alpha]_D^{27}$ +28 (*c* 2.0 in $CHCl_3$)); ν_{max} (neat)/cm^{-1} 3600-3300w br, 1652m (C=O)^{ketone},

1594m, 1510m, 1453m, 1440m, 1348m, 1299m, 1275m, 1214m, 1196m, 1155m, 1090m, 1005m, 916m, 829m; δ_H (500 MHz, $CDCl_3$) 1.73 (3H, s, C(4')H_3), 1.80 (3H, s, C(5')H_3), 3.36 (1H, dd, *J* 7.5, 16.0 Hz, C(1')HH'), 3.45 (1H, dd, *J* 7.5, 16.0 Hz, C(1')HH'), 3.76 (3H, s, C(2'')H_3), 3.81 (3H, s, C(3'')H_3), 3.83 (1H, d, *J* 4.0 Hz, C(12a)H), 4.18 (1H, d, *J* 12.0 Hz, C(6)HH'), 4.62 (1H, dd, *J* 3.0, 12.0 Hz, C(6)HH'), 4.91 (1H, dd, *J* 3.0, 4.0 Hz, C(6a)H), 5.21 (1H, t, *J* 7.5 Hz, C(2')H), 6.06 (1H, s, C(9)OH), 6.44 (1H, s, C(4)H), 6.50 (1H, d, *J* 8.5 Hz, C(10)H), 6.78 (1H, s, C(1)H), 7.76 (1H, d, *J* 8.5 Hz, C(11)H); δ_C (125 MHz, $CDCl_3$) 17.9 (C(5')H_3), 22.1 (C(1')H_2), 25.8 (C(4')H_3), 44.2 (C(12a)H), 55.8 (C(3'')H_3), 56.3 (C(2'')H_3), 66.3 (C(6)H_2), 72.2 (C(6a)H), 100.9 (C(4)H), 104.7 (C(13)), 110.4 (C(1)H), 110.9 (C(10)H), 113.0 (C(8)), 114.1 (C(11a)), 120.8 (C(11)H), 127.2 (C(2')H), 135.7 (C(3')), 143.8 (C(2)), 147.5 (C(3)), 149.4 (C(4a)), 159.8 (C(7a)), 161.8 (C(9)), 189.8 (C(12)); LRMS *m/z* found 397.2, $C_{23}H_{25}O_6$ $[M+H]^+$ requires 397.2; HRMS *m/z* found 419.1464, $C_{23}H_{24}O_6Na$ $[M+Na]^+$ requires 419.1471.

(6a*S*,12a*S*)-Deguelin 1

Phenylselenyl chloride (1.06 g, 5.56 mmol) was added to a solution of (6a*S*,12a*S*)-rot-2'-enonic acid **5** (2.00 g, 5.05 mmol) in anhydrous dichloromethane (80.0 mL) under an atmosphere of nitrogen at -40 ^oC. The reaction mixture was stirred for 2 h at -40 ^oC, then warmed to room temperature and stirred for an additional 1 h. The mixture was concentrated *in vacuo* and the crude yellow foam was dissolved in THF (80.0 mL) and cooled to 0 ^oC. Hydrogen peroxide (1.14 mL, 30 % aqueous solution, approx. 10 mmol) was then added and the reaction mixture was stirred at 0 ^oC for 1 h, warmed to room temperature and stirred for an additional 18 h. The mixture was extracted into diethyl ether (80 mL), washed with 5 % aqueous sodium bicarbonate solution (3 x 80 mL), water (80 mL) and brine (80 mL), dried over anhydrous $MgSO_4$ and concentrated *in vacuo*. The crude yellow foam obtained was purified by flash column chromatography (hexane-ethyl acetate; 3:1 then 2:1) to afford (6a*S*,12a*S*)-deguelin **1** as a bright yellow oil that solidified upon scratching to form a bright yellow amorphous powder (1.61 g, 81%). R_f 0.30 (hexane-ethyl acetate; 2:1); $[\alpha]_D^{20}$ -94 (*c* 0.1 in $CHCl_3$); v_{max} (neat)/cm^{-1} 1671m $(C=O)^{ketone}$, 1635w, 1596m, 1577m, 1511m, 1440m, 1344m, 1273m, 1212m, 1197m, 1110m, 1093m, 1009m, 819m; δ_H (500 MHz, $CDCl_3$) 1.38 (3H, s, C(7')H_3), 1.45 (3H, s, C(8')H_3), 3.77 (3H, s, C(2')H_3), 3.81 (3H, s, C(3')H_3), 3.84 (1H, d, *J* 4.5 Hz, C(12a)H), 4.19 (1H, d, *J* 12.0 Hz, C(6)HH'), 4.64 (1H, dd, *J* 3.0, 12.0 Hz, C(6)HH'), 4.92 (1H, dd, *J* 3.0, 4.5 Hz, C(6a)H), 5.56 (1H, d, *J* 10.0 Hz, C(5')H), 6.45 (1H, d, *J* 8.5 Hz, C(10)H), 6.46 (1H, s, C(4)H), 6.65 (1H, d, *J* 10.0 Hz, C(4')H), 6.79 (1H, s, C(1)H), 7.74 (1H, d, *J* 8.5 Hz, C(11)H); δ_C (125 MHz, $CDCl_3$) 28.1 (C(7')H_3), 28.5 (C(8')H_3), 44.4 (C(12a)H), 55.8 (C(3')H_3), 56.3 (C(2')H_3), 66.3 (C(6)H_2), 72.4 (C(6a)H), 77.7 (C(6')), 100.9 (C(4)H), 104.7 (C(13)), 100.9 (C(8)), 110.4 (C(1)H), 111.5 (C(10)H), 112.7 (C(11a)), 115.7 (C(4')H), 128.5 (C(11)H), 128.7 (C(5')H),

143.3 (C(2)), 147.4 (C(3)), 149.4 (C(4a)), 156.9 (C(7a)), 160.1 (C(9)), 189.2 (C(12)); LRMS *m/z* found 395.1, $C_{23}H_{23}O_6$ $[M+H]^+$ requires 395.1; HRMS *m/z* found 417.1300, $C_{23}H_{22}O_6Na$ $[M+Na]^+$ requires 417.1314.

(6a*R*,12a*R*)-Tephrosin 2

A solution of potassium dichromate (842 mg, 2.84 mmol) in water (12.0 mL) was added dropwise over a period of 10 min to a solution of (6a*S*,12a*S*)-deguelin **1** (1.20 g, 3.05 mmol) in acetic acid (24.0 mL) at 60 ^oC. The reaction mixture was stirred at 60 ^oC for a further 0.5 h then cooled to room temperature and stirred for an additional 18 h. Ice-cold water (200 mL) was then added and the mixture was stirred vigorously for 0.5 h while a yellow precipitate formed, which was collected by filtration and dried *in vacuo* overnight. The precipitate was purified by flash column chromatography (hexane- ethyl acetate; 2:1) to afford (6a*R*,12a*R*)-tephrosin **2** as a bright yellow amorphous solid (962 mg, 76%). R_f 0.30 (hexane-ethyl acetate; 1:1); $[\alpha]_D^{20}$ -88 (*c* 0.1 in $CHCl_3$); ν_{max} (neat)/cm^{-1} 3600-3300w br, 1674m $(C=O)^{ketone}$, 1596m, 1577m, 1509m, 1441m, 1330m, 1270m, 1218m, 1200m, 1159m, 1109m, 1087m, 1027m, 884m, 816m; δ_H (500 MHz, $CDCl_3$) 1.39 (3H, s, C(7')H_3), 1.45 (3H, s, C(8')H_3), 3.73 (3H, s, C(2')H_3), 3.81 (3H, s, C(3')H_3), 4.40 (1H, s, C(12a)OH), 4.49 (1H, dd, *J* 1.0, 12.0 Hz, C(6)HH'), 4.57 (1H, dd, *J* 1.0, 2.5 Hz, C(6a)H), 4.63 (1H, dd, *J* 2.5, 12.0 Hz, C(6)HH'), 5.55 (1H, d, *J* 10.0 Hz, C(5')H), 6.47 (1H, d, *J* 9.0 Hz, C(10)H), 6.48 (1H, s, C(4)H), 6.56 (1H, s, C(1)H), 6.60 (1H, d, *J* 10.0 Hz, C(4')H), 7.72 (1H, d, *J* 11.0 Hz, C(11)H); δ_C (125 MHz, $CDCl_3$) 28.3 (C(7')H_3), 28.5 (C(8')H_3), 55.9 (C(3')H_3), 56.3 (C(2')H_3), 63.8 (C(6)H_2), 67.4 (C(12a)), 76.2 (C(6a)H), 78.0 (C(6')), 101.0 (C(4)H), 108.6 (C(13)), 109.1 (C(8)), 109.3 (C(1)H), 111. (C(11a)), 111.9 (C(10)H), 115.4 (C(4')H), 128.5 (C(11)H), 128.8 (C(5')H), 143.9 (C(2)), 148.4 (C(3)), 151.1 (C(4a)), 156.6 (C(7a)), 160.8 (C(9)), 191.4 (C(12)); LRMS *m/z* found 393.1, $C_{23}H_{22}O_6$ $[M-H_2O+H]^+$ requires 393.1; HRMS *m/z* found 433.1262, $C_{23}H_{22}O_7Na$ $[M+Na]^+$ requires 433.1258.

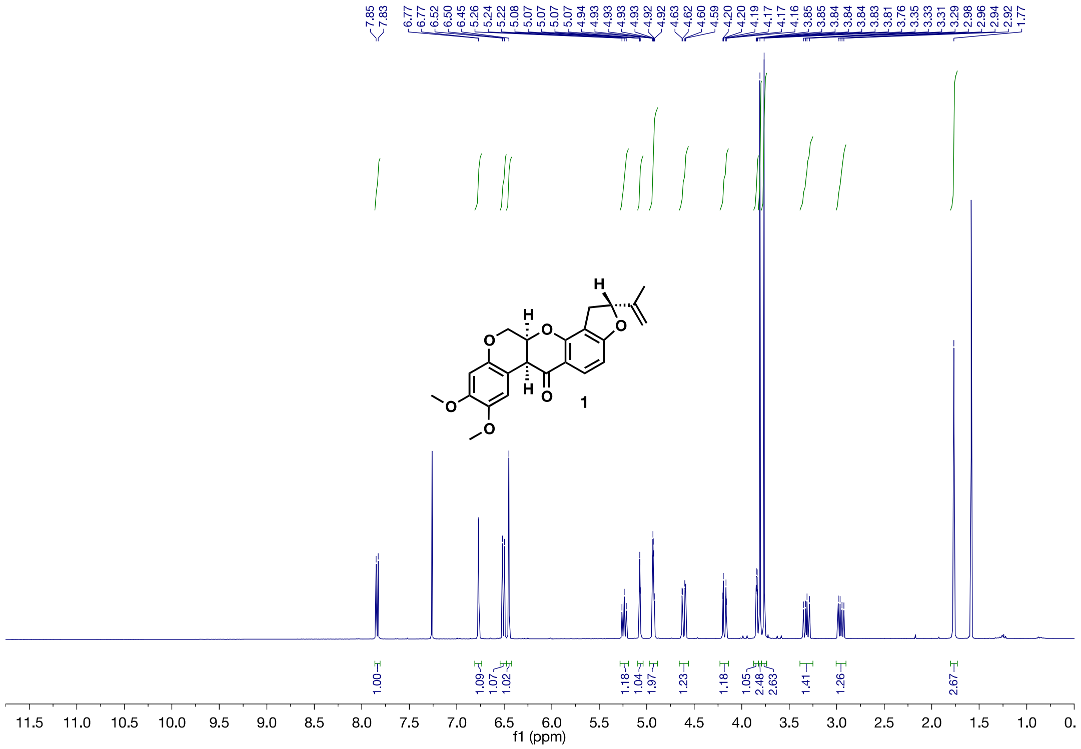
7.85
7.83
6.77
6.77
6.52
6.50
6.45
5.26
5.24
5.22
5.08
5.07
5.07
5.07
5.07
4.94
4.93
4.93
4.93
4.93
4.92
4.92
4.63
4.62
4.60
4.59
4.20
4.20
4.19
4.17
4.17
4.16
3.85
3.85
3.84
3.84
3.84
3.83
3.81
3.76
3.35
3.33
3.31
3.29
2.98
2.96
2.94
2.92
1.77
H
O
1
1.00
1.09
1.07
1.02
1.18
1.04
1.97
1.23
1.18
1.05
2.48
2.63
1.41
1.26
2.67
11.5 11.0 10.5 10.0 9.5 9.0 8.5 8.0 7.5 7.0 6.5 6.0 5.5 5.0 4.5 4.0 3.5 3.0 2.5 2.0 1.5 1.0 0.
f1 (ppm)

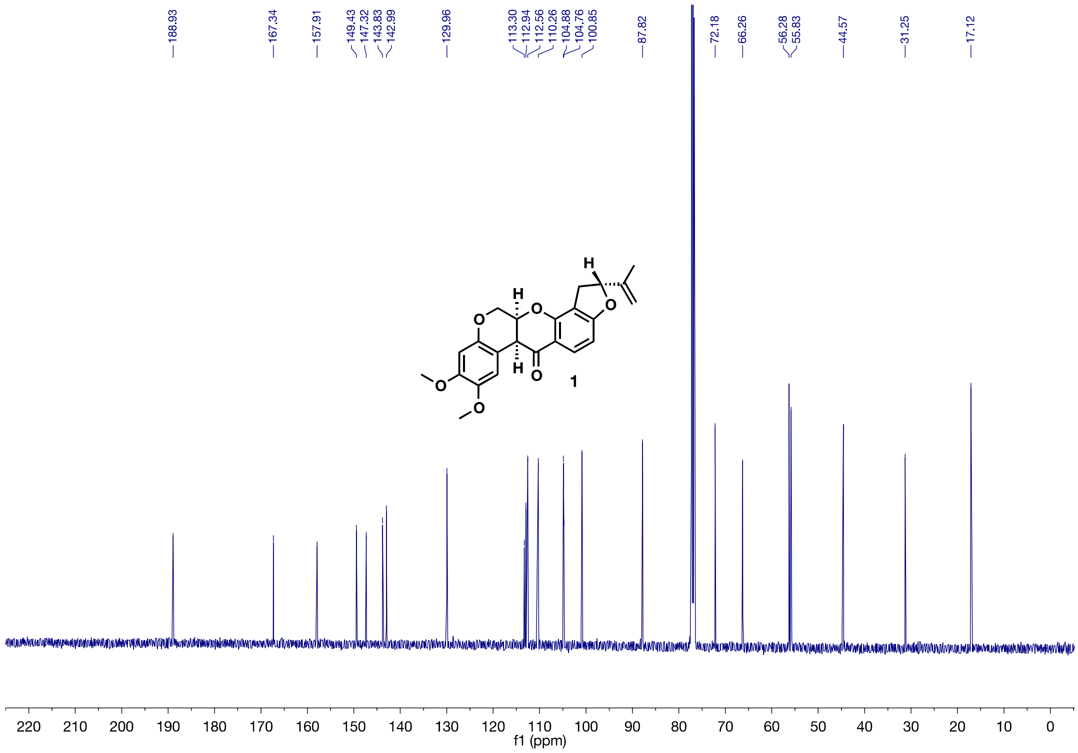
188.93
167.34
157.91
149.43
147.32
143.83
142.99
129.96
113.30
112.94
112.56
110.26
104.88
104.76
100.85
87.82
72.18
66.26
56.28
55.83
44.57
31.25
17.12
H
O
1
220 210 200 190 180 170 160 150 140 130 120 110 100 90 80 70 60 50 40 30 20 10 0
f1 (ppm)

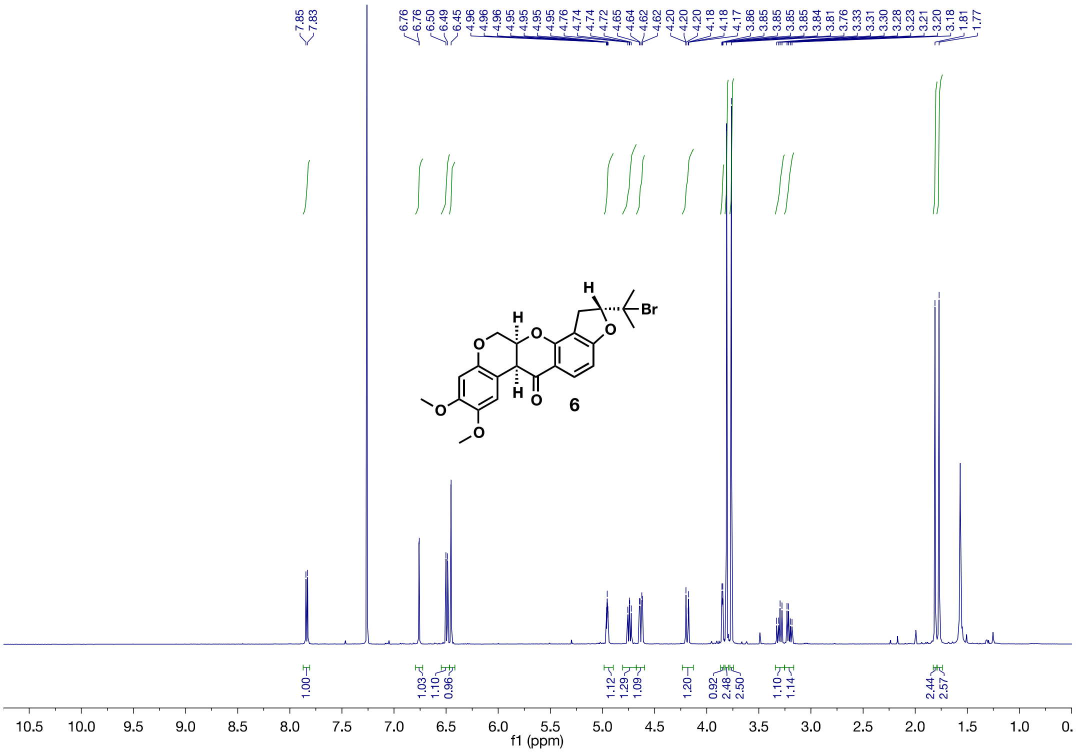
7.85
7.83
6.76
6.76
6.50
6.49
6.45
4.96
4.96
4.95
4.95
4.95
4.95
4.76
4.74
4.74
4.72
4.65
4.64
4.62
4.62
4.20
4.20
4.18
4.18
4.17
3.86
3.85
3.85
3.85
3.84
3.81
3.76
3.33
3.31
3.30
3.28
3.23
3.21
3.20
3.18
1.81
1.77
H
Br
O
O
O
H
H
O
O
O
6
1.00
1.03
1.10
0.96
1.12
1.29
1.09
1.20
0.92
2.48
2.50
1.10
1.14
2.44
2.57
10.5 10.0 9.5 9.0 8.5 8.0 7.5 7.0 6.5 6.0 5.5 5.0 4.5 4.0 3.5 3.0 2.5 2.0 1.5 1.0 0.
f1 (ppm)

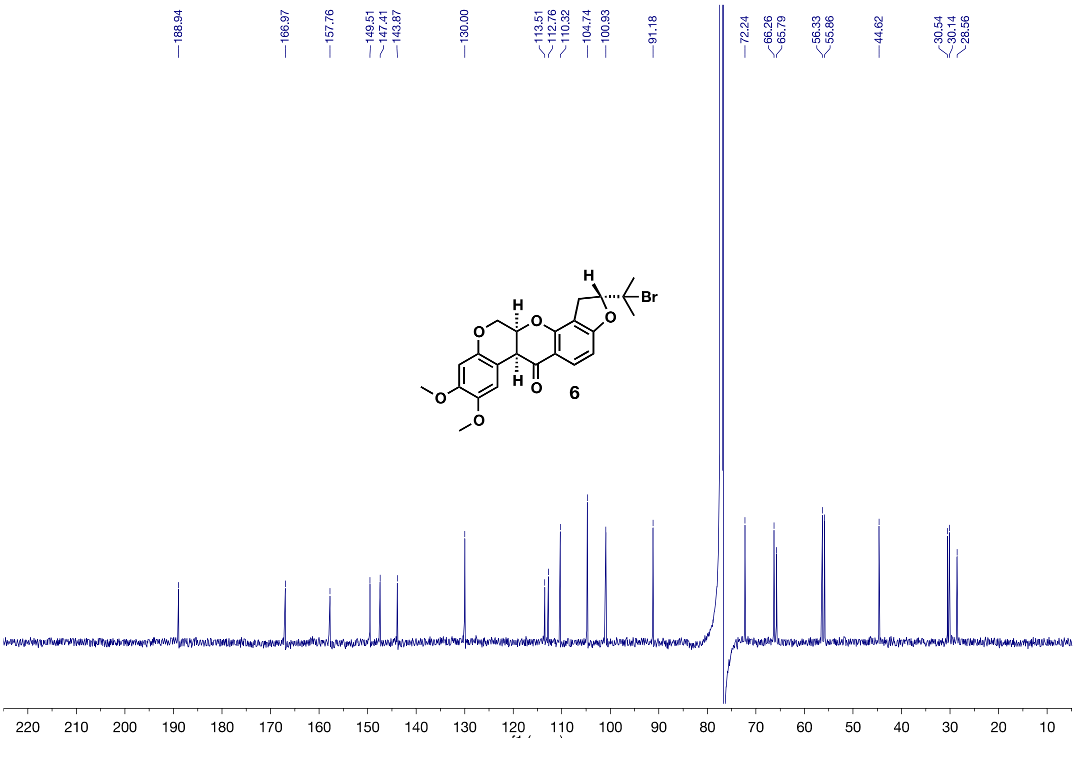
188.94
166.97
157.76
149.51
147.41
143.87
130.00
113.51
112.76
110.32
104.74
100.93
91.18
72.24
66.26
65.79
56.33
55.86
44.62
30.54
30.14
28.56
H
Br
O
O
O
H
H
O
O
O
6
220 210 200 190 180 170 160 150 140 130 120 110 100 90 80 70 60 50 40 30 20 10

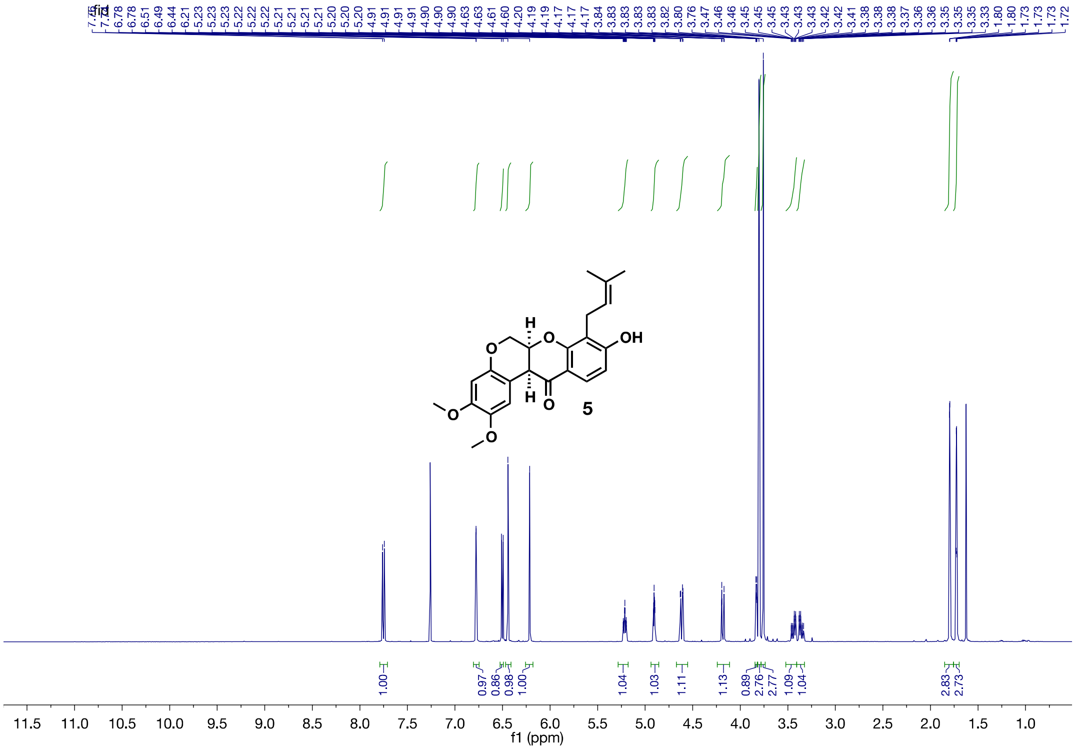
OH
O
H
5
f1 (ppm)

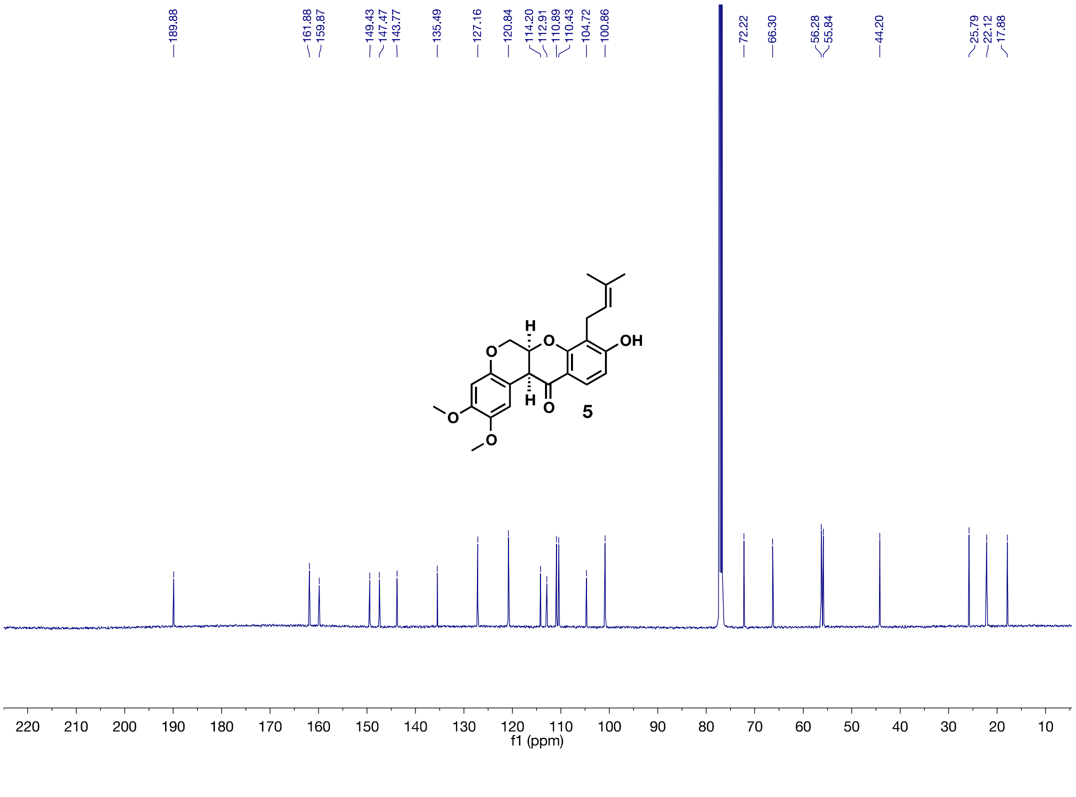
189.88
161.88
159.87
149.43
147.47
143.77
135.49
127.16
120.84
114.20
112.91
110.89
110.43
104.72
100.86
72.22
66.30
56.28
55.84
44.20
25.79
22.12
17.88
OH
O
H
5
f1 (ppm)

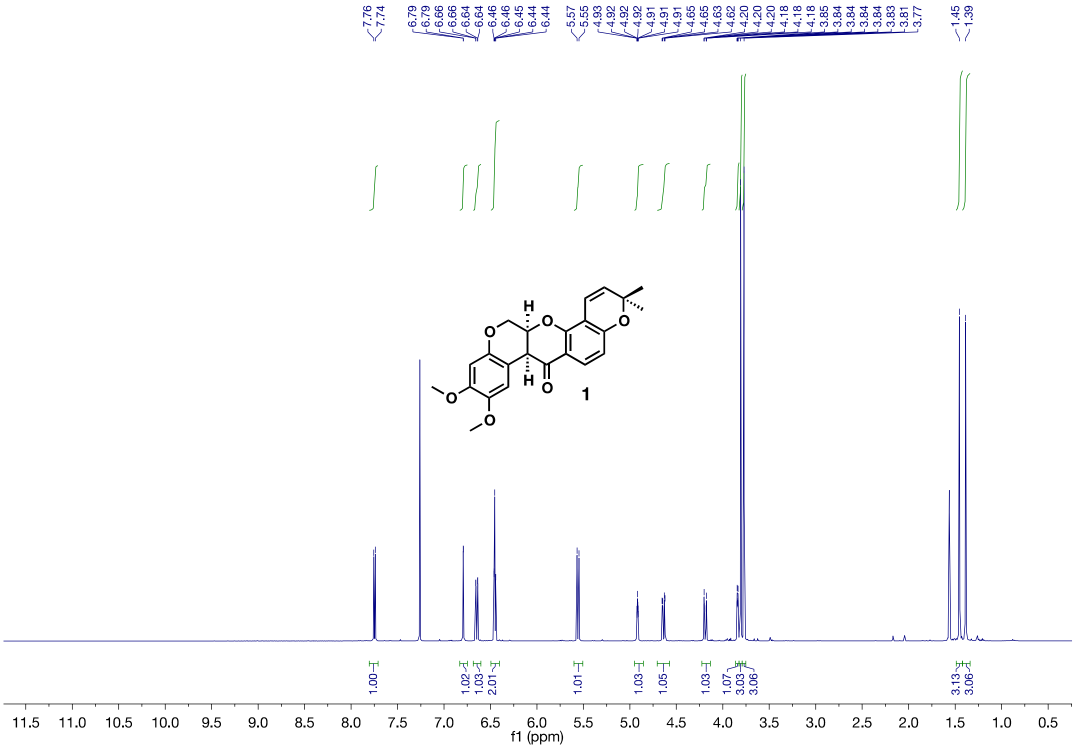
7.76
7.74
6.79
6.79
6.66
6.66
6.64
6.64
6.46
6.46
6.45
6.44
6.44
5.57
5.55
4.93
4.92
4.92
4.92
4.91
4.91
4.91
4.65
4.63
4.62
4.20
4.20
4.20
4.18
4.18
3.85
3.84
3.84
3.84
3.83
3.81
3.77
1.45
1.39
H
O
O
O
H
O
O
O
1
1.00
1.02
1.03
2.01
1.01
1.03
1.05
1.03
1.07
3.03
3.06
3.13
3.06
11.5 11.0 10.5 10.0 9.5 9.0 8.5 8.0 7.5 7.0 6.5 6.0 5.5 5.0 4.5 4.0 3.5 3.0 2.5 2.0 1.5 1.0 0.5
f1 (ppm)

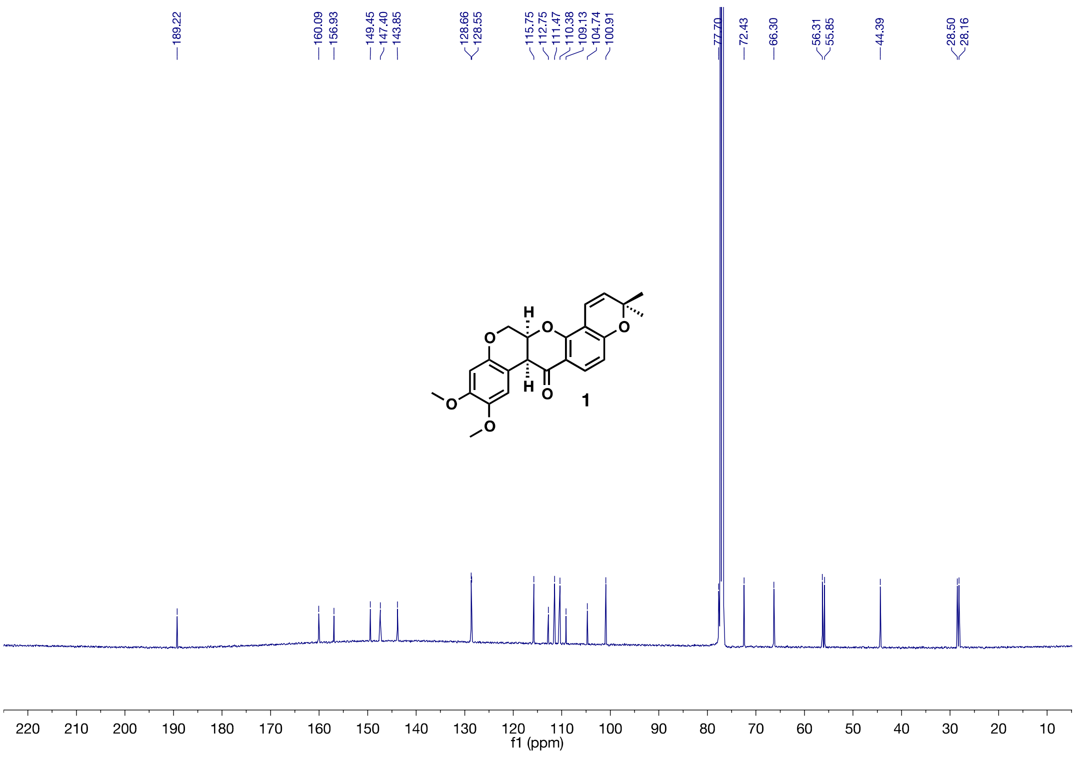
189.22
160.09
156.93
149.45
147.40
143.85
128.66
128.55
115.75
112.75
111.47
110.38
109.13
104.74
100.91
77.70
72.43
66.30
56.31
55.85
44.39
28.50
28.16
H
O
O
O
H
O
O
O
1
220 210 200 190 180 170 160 150 140 130 120 110 100 90 80 70 60 50 40 30 20 10
f1 (ppm)

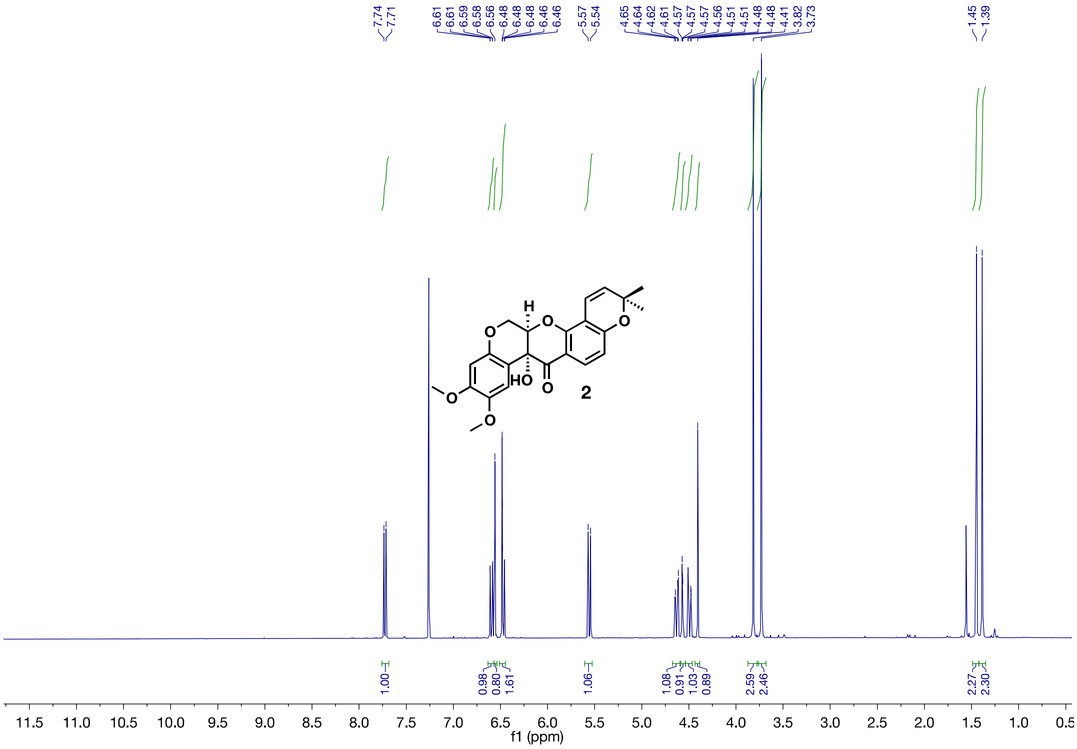
7.74
7.71
6.61
6.61
6.59
6.58
6.56
6.48
6.48
6.48
6.46
6.46
5.57
5.54
4.65
4.64
4.62
4.61
4.57
4.57
4.57
4.56
4.51
4.51
4.48
4.48
4.41
3.82
3.73
1.45
1.39
HO
O
H
2
1.00
0.98
0.80
1.61
1.06
1.08
0.91
1.03
0.89
2.59
2.46
2.27
2.30
11.5 11.0 10.5 10.0 9.5 9.0 8.5 8.0 7.5 7.0 6.5 6.0 5.5 5.0 4.5 4.0 3.5 3.0 2.5 2.0 1.5 1.0 0.5
f1 (ppm)

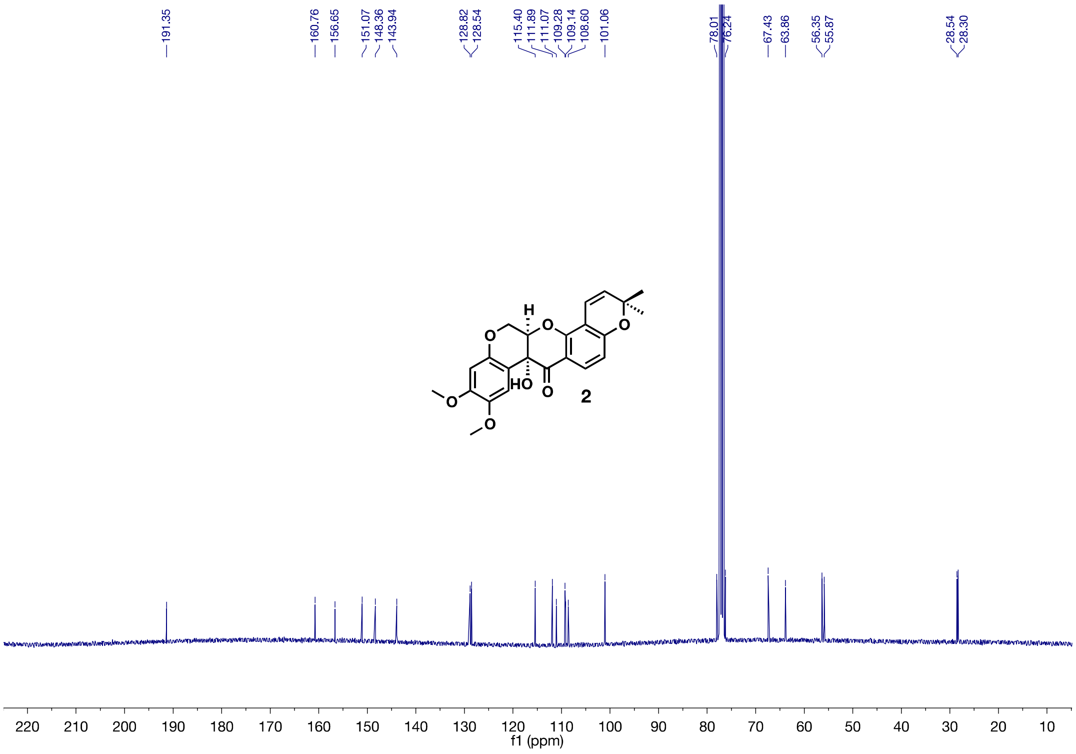
191.35
160.76
156.65
151.07
148.36
143.94
128.82
128.54
115.40
111.89
111.07
109.28
109.14
108.60
101.06
78.01
76.24
67.43
63.86
56.35
55.87
28.54
28.30
HO
O
H
2
220 210 200 190 180 170 160 150 140 130 120 110 100 90 80 70 60 50 40 30 20 10
f1 (ppm)

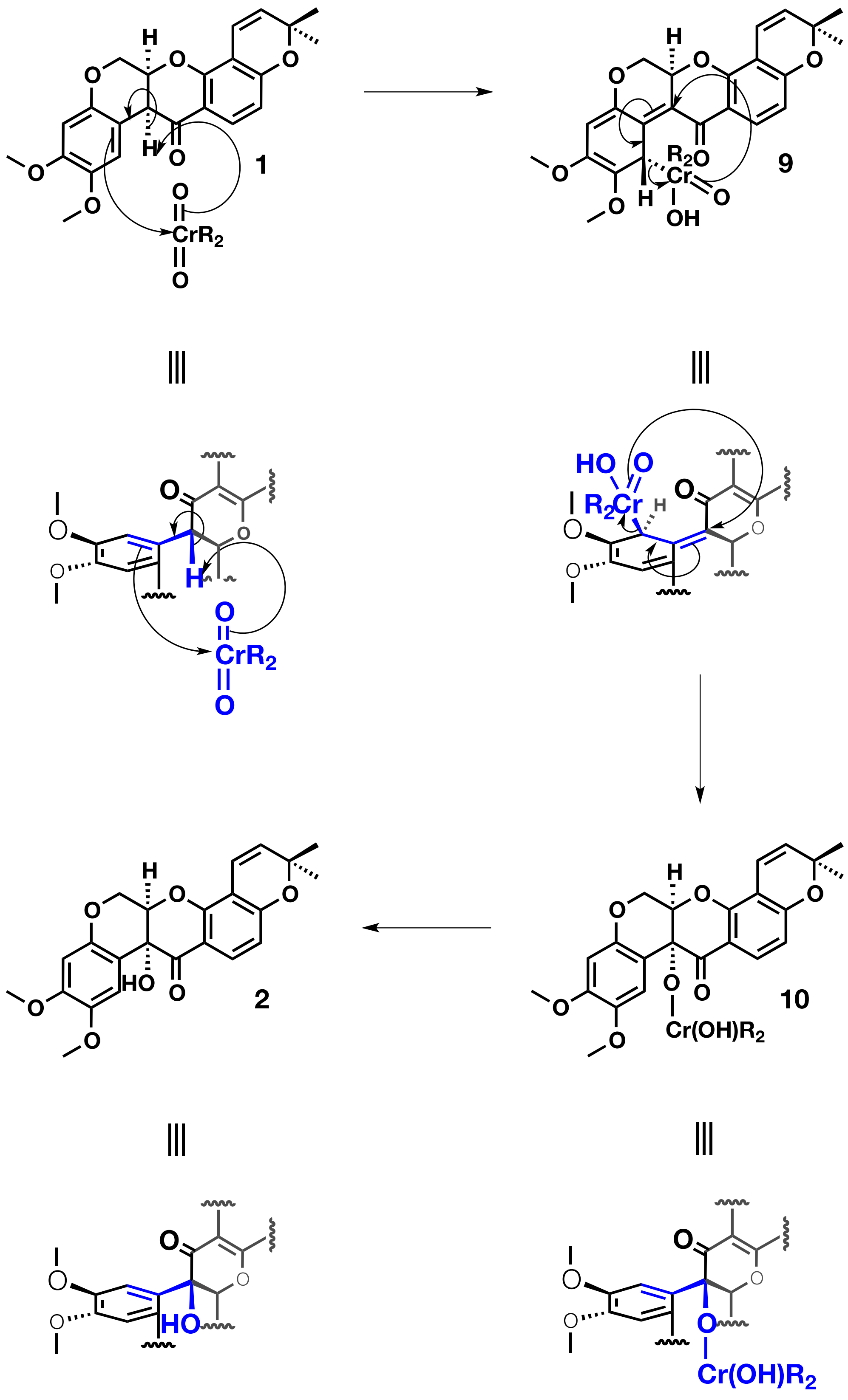

ESI Figure 2: An alternative version of Scheme 2 with drawings of the 3-D conformations of the partial structures of molecules **1**, **2**, **9** and **10**.
